# Perceptions of scientific research literature and strategies for reading papers depend on academic career stage

**DOI:** 10.1371/journal.pone.0189753

**Published:** 2017-12-28

**Authors:** Katharine E. Hubbard, Sonja D. Dunbar

**Affiliations:** Department of Plant Sciences, University of Cambridge, Cambridge, United Kingdom; Waseda University, JAPAN

## Abstract

Reading primary research literature is an essential skill for all scientists and students on science degree programmes, however little is known about how researchers at different career stages interact with and interpret scientific papers. To explore this, we conducted a survey of 260 undergraduate students and researchers in Biological Sciences at a research intensive UK university. Responses to Likert scale questions demonstrated increases in confidence and skill with reading the literature between individuals at each career stage, including between postdoctoral researchers and faculty academics. The survey indicated that individuals at different career stages valued different sections of scientific papers, and skill in reading the results section develops slowly over the course of an academic career. Inexperienced readers found the methods and results sections of research papers the most difficult to read, and undervalued the importance of the results section and critical interpretation of data. These data highlight a need for structured support with reading scientific literature at multiple career stages, and for senior academics to be aware that junior colleagues may prioritise their reading differently. We propose a model for the development of literature processing skills, and consider the need for training strategies to help inexperienced readers engage with primary literature, and therefore develop important skills that underpin scientific careers. We also encourage researchers to be mindful of language used when writing papers, and to be more inclusive of diverse audiences when disseminating their work.

## Introduction

Engaging with the scientific literature is a key skill for researchers and students on scientific degree programmes; it has been estimated that scientists spend 23% of total work time reading [1,2]. The number of papers an individual scientist reads annually increased from 188 to 280 between 1993 and 2005, while total time spent only increased marginally [3]. Scientific writing is characterised by highly specialist vocabulary, concise and precise use of language, often accompanied by complex grammatical structures [4,5]. Making sense of scientific papers can be therefore cognitively challenging, particularly for readers who may be unfamiliar with the terminology of the field [6]. This challenge is faced by undergraduate students and early career scientists, but may also be encountered by experienced researchers exploring the literature in another discipline. We currently have a relatively poor understanding of how skills relating to the processing of scientific text develop through academic careers, the potential barriers to engaging with technical documentation and subsequent impact on the development of disciplinary or interdisciplinary research activities.

Most university level science courses require undergraduate students to engage with primary research literature, which is not generally used in school level education. In Biological Sciences, research literature is typically introduced early on or part way through the programme of study, with an expectation that students will be highly engaged with the literature in their final year of study. For example, the Quality Assurance Agency for UK degrees states that Biosciences graduates should have “*the ability to read and use appropriate literature with a full and critical understanding*, *while addressing such questions as content*, *context*, *aims*, *objectives*, *quality of information*, *and its interpretation and application*.” [7]. Postgraduate students are generally expected to be fully engaged in primary literature throughout their graduate programmes; some postgraduate training programmes also include support for reading and interpreting literature, but many institutions assume this is a skill developed at undergraduate level. Some disciplines (e.g. theoretical physics) rely less heavily on primary research papers at undergraduate level due to their complexity, but all early career researchers will encounter scientific literature at some point in their training.

There have been many strategies and supporting resources developed to help inexperienced readers engage with the primary literature [6,8–14]. Undergraduates using these approaches have significantly increased performance in critical thinking tests, and report increased interest in primary research when interviewed after being taught specific reading strategies [11,15]. Small group journal clubs, with dedicated academic mentors, were shown to increase both scientific literacy and confidence in communicating scientifically with academic colleagues, ultimately facilitating transition to postgraduate study [16]. It is suggested that such strategies could help alleviate disengagement with science and prevent students dropping out of STEM subjects, particularly those from underrepresented backgrounds [11].

Many of these ‘strategy’ papers rely on anecdotal evidence that undergraduates adopt superficial reading strategies and lack analytical skills [17], and make a tacit assumption that more experienced researchers read the literature ‘correctly’. Faculty members are assumed to be ‘experts’ possessing both good content knowledge and science processing skills [18]. The skills that distinguish experts from novices stem from having a conceptual framework that allows experts to organise content effectively, combine it with relevant knowledge easily and recognise meaningful patterns within the information presented [19]. While the distinction between novice and expert is clear, the career stage at which the transition to expert is completed is broadly unknown.

Here we present results of a survey of researchers and students within Biological Sciences at a single research-intensive institution in the UK. This study aimed to begin an exploration of attitudes towards reading the literature, and start to uncover how readers at different career stages approached scientific papers. To explore this with participants who are likely to read a large number of scientific papers, the survey was administered at the University of Cambridge (UK), which has high academic entry standards for both undergraduate and postgraduate students. Survey participants include undergraduate students, PhD students, postdoctoral researchers and academic researchers, representing a wide range of career stages.

## Methods

### Survey design, implementation and ethics

A pilot survey of both students and researchers was designed to answer the following lines of enquiry for both students and researchers:

1. How frequently did participants read scientific papers?
2. How did participants feel about reading scientific papers?
3. How easy to read did participants find different sections of papers?
4. How important did participants think different sections of papers were?
5. [Students only] How much training had they had in reading papers?
6. [Researchers only] What advice would they give to someone reading a paper for the first time.

As a result of the pilot study, a full survey was designed to follow similar lines of inquiry. Where appropriate, this survey asked about primary research papers and review papers separately. This survey also included questions about the search engines used to find papers as an additional line of enquiry. Two versions of the full survey were designed, one for undergraduate students and one for postgraduate researchers. Questions were either direct replicas, designed to be mirrored where appropriate, or were unique to one of the two groups (see S1 and S2 Files for the text of the questionnaires). An optional ‘teaching’ section was added to the researchers survey to explore approaches to training students to read the literature, but the results of this section are outside the scope of the current study and are not included here. The results presented here are collected from the full survey, and do not include the results of the initial pilot questionnaire.

The undergraduate survey was administered at the end of the academic year (i.e. after examinations) to 2nd year and 3rd (final) year undergraduates studying biological sciences courses on the Natural Sciences programme. These courses included Biochemistry and Molecular Biology, Cell and Developmental Biology, Ecology, Genetics, Neuroscience, Pathology, Physiology, Plant Sciences, Psychology and Zoology. The researcher survey was administered at the same time to all researchers in biological sciences departments, which included Biochemistry, Genetics, Pathology, Physiology Development and Neuroscience, Plant Sciences, Psychology and Zoology. All participants were invited to participate via a weblink taking them to the online survey, which was hosted by www.surveymonkey.com.

No appropriate institutional ethics committee for educational research was in place at the time of the study being completed, however the study was designed in accordance with BERA (British Educational Research Association) ethical guidelines. At the start of the questionnaire, participants were presented with a short description of the study and the way in which their data would be stored and used. At the end of the survey participants were asked for their informed consent to their answers being used in publications resulting from the work, and to provide their email address if they were willing to participate in a follow-up interviews. Email addresses were removed from spreadsheets prior to data analysis, and responses remained anonymous throughout.

### Quantitative analysis

Responses to Likert Scale questions were converted to numbers from 1–6 for statistical analysis, with 1 representing the most negative response to the question (e.g. Strongly disagree) and 6 the most positive (e.g. Strongly agree). The ordinal nature of the questionnaire data meant that parametric statistics were inappropriate for analysis, so non-parametric tests are used throughout with alpha = 0.95. Observations were independent, with no individuals belonging to more than one career group. For comparisons of multiple groups Kruskal-Wallis H tests were used, followed by pairwise Mann-Whitney U posthoc tests between particular groups where appropriate. Actual P values are reported throughout, except where P < 0.001. Statistical significance is indicated by asterisks throughout the manuscript; ** indicates P<0.01, * indicates P<0.05. For analysis of differences in responses between disciplines participants were grouped as ‘Molecular’, ‘Ecological’ or ‘Other’ on the basis of their reported subject or research area.

### Qualitative analysis

For the question If you could only give one piece of advice to someone reading a scientific paper for the first time, what would it be?” responses were collected as free text. Responses were copied directly into NVivo and then assigned nodes to represent initial coding. Nodes were then aggregated into major themes, and the number of mentions of each theme determined. Themes or subthemes with fewer than 5 mentions out of the 88 responses are not presented for clarity.

Word clouds were generated using the ‘wordcloud’ package in R. Free text responses to the question were compiled into a.txt file, converted to lowercase and punctuation marks removed. Commonly used words in English were removed, along with the words ‘read’, ‘reading’ and ‘papers’. Words being mentioned fewer than 5 times are not presented for clarity, and colours used do not represent any formal analysis.

## Results

We obtained 300 responses to the survey, 260 of which completed responses to all questions. Of those who answered at least one question in the survey, participants included undergraduates in their second (*n* = 81) and third (final) year of study (*n* = 66), PhD students (*n* = 53), PostDoctoral researchers (*n* = 68) and Academics (*n* = 49). Participants came from a range of disciplines within biological sciences, including biochemistry, physiology, ecology and mathematical biology (see S4 File for detailed breakdown). For the undergraduate survey, response rates were 47% for second years and 42% for third years. We were unable to determine response rates for the research survey as we did not have access to the total number of researchers employed by the university.

### Perceptions of the research literature changes throughout academic careers

**Fig 1:**
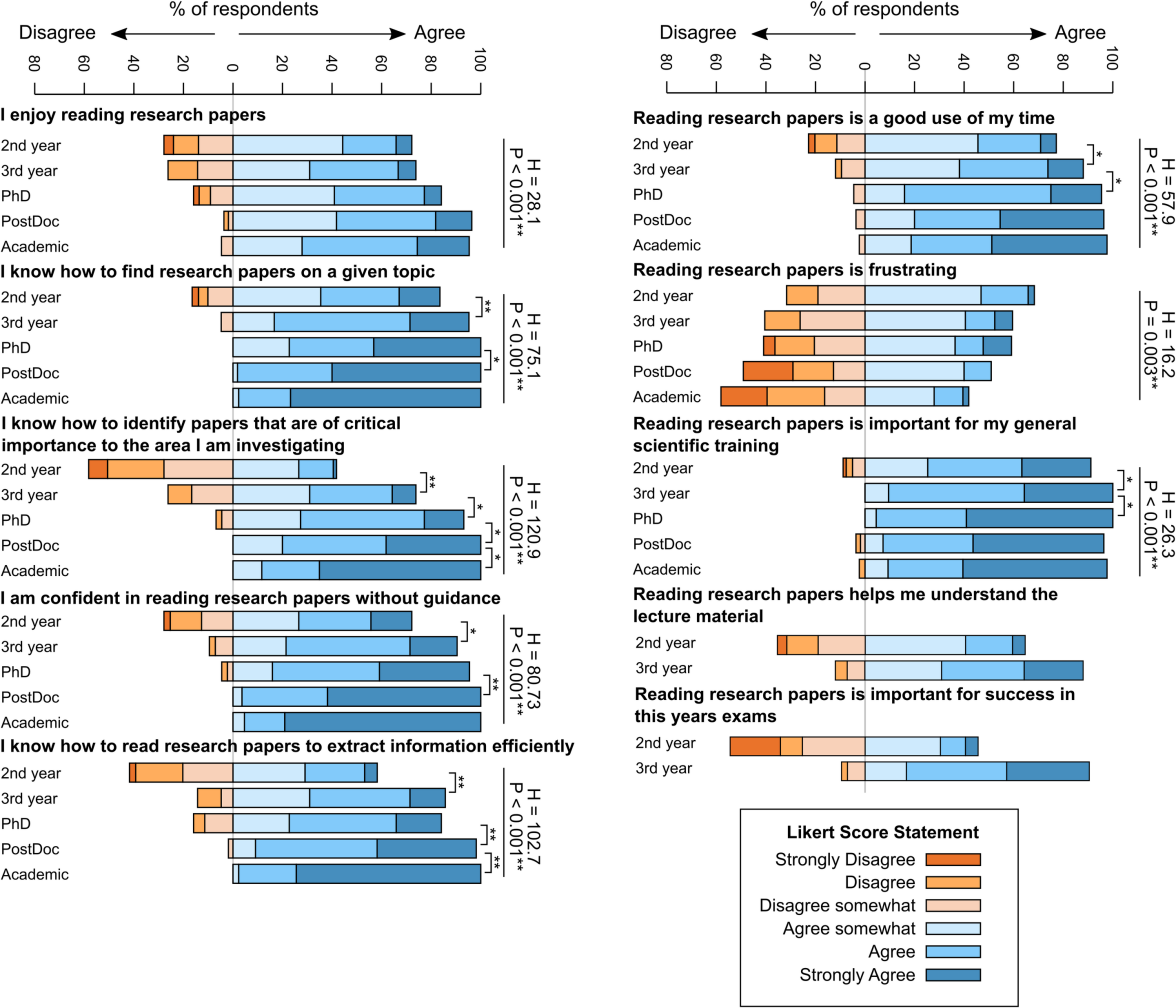
The relationship with the research literature changes as a function of career stage. Responses to the 6 point Likert scale questions for 2nd year undergraduate n = 79; 3rd year undergraduate n = 42; PhD student n = 44; PostDoctoral researcher n = 55; Academic n = 43. Statistics alongside straight lines indicate the results of Kruskal-Wallis H tests (Kruskal-Wallis H and P values presented, degrees of freedom = 4 in all cases). Square brackets indicate significant Mann-Whitney tests for differences between groups, ** indicates P<0.01, * indicates P<0.05.

To explore the relationship that participants had with the literature, we asked them to what extent they agreed with a series of statements on a 6 point Likert scale (Fig 1). The statement with the highest level agreement with all career stages was ‘Reading research papers is important for my general scientific training’, where only 10 respondents had any level of disagreement with the statement. The statement that was most disagreed with was the negatively worded question ‘Reading research papers is frustrating’, where 112 of the 300 participants disagreed (37%). Interestingly, 42% of academics agreed that they found reading papers frustrating, indicating that even experienced readers encountered difficulties in engaging with the primary research literature.

For all 8 statements there was a significant difference in the level agreement with the statement across the five career stages, with attitudes towards the literature generally becoming more positive with each subsequent stage. For the statement ‘I enjoy reading research papers’, attitudes became more positive a function of experience; 72% (57 out of 79) of 2nd year students agreed with the statement to some extent, whereas 95% of academics (41 out of 43) agreed that they enjoyed reading research papers. While this difference across all five stages was significant (Kruskal-Wallis H = 28.1, d.f. = 4, P < 0.001**), there were no significant differences between subsequent stages (e.g. PhD students were not significantly more likely to agree with the statement than 3rd year undergraduates), suggesting increasing enjoyment of the literature was not associated with particular career transitions. Undergraduates generally agreed that reading research papers was important for understanding material covered in lectures. 90% of 3^rd^ year undergraduates agreed that reading research papers was important for success in their end of year exams, but only 45% of 2^nd^ years agreed with this statement. In contrast, there was no difference in responses to these questions as a function of biological discipline (e.g. ‘I enjoy reading papers’ H = 0.68, d.f. = 2, P = 0.71; ‘I am confident in reading research papers without guidance’ H = 0.561. d.f. = 2, P = 0.561), indicating that ease with the literature not significantly influenced by disciplinary conventions.

The level of agreement with the statements increased significantly between sequential career stages, indicating that some or all career transitions were important in developing a relationship with the literature, not just at the undergraduate to postgraduate stage. For example, for the statement ‘I know how to identify papers that are of critical importance to the area I am investigating’, there was a significant increase in the level of agreement with the statement at every career stage, including between PostDoctoral Researchers and Academics (Mann-Whitney U = 866.5, P = 0.013*). A similar pattern was present for the statement ‘I know how to read research papers to extract information efficiently’, where PostDocs and Academics also had significantly different levels of agreement (U = 758.5, P = 0.001**), although for this statement there were no differences between 3rd year undergraduates and PhD students (U = 859.0, P = 0.557).

Perceptions of the literature may be shaped by the level of familiarity participants had with scientific papers. We therefore determined how many papers participants at different career stages were reading. All researchers and 3rd year undergraduates reported reading primary research papers, and only 7% of 2nd years said that they had not read any research papers, meaning that the vast majority of participants were engaging with primary literature from at least one source. More senior researchers were more likely to report reading multiple papers per week, with many academics reporting reading multiple research papers per day.

### Undergraduates and early career researchers find methods and results sections the most difficult to understand

To explore where the challenges of engaging with the literature were in more detail, we asked survey participants “How easy do you usually find it to understand the following aspects of a research paper?”, with responses being recorded on a 6 point Likert scale. The figures and tables of the results section were considered in a separate question to the text-based description of the results to allow these aspects to be considered independently.

**Fig 2:**
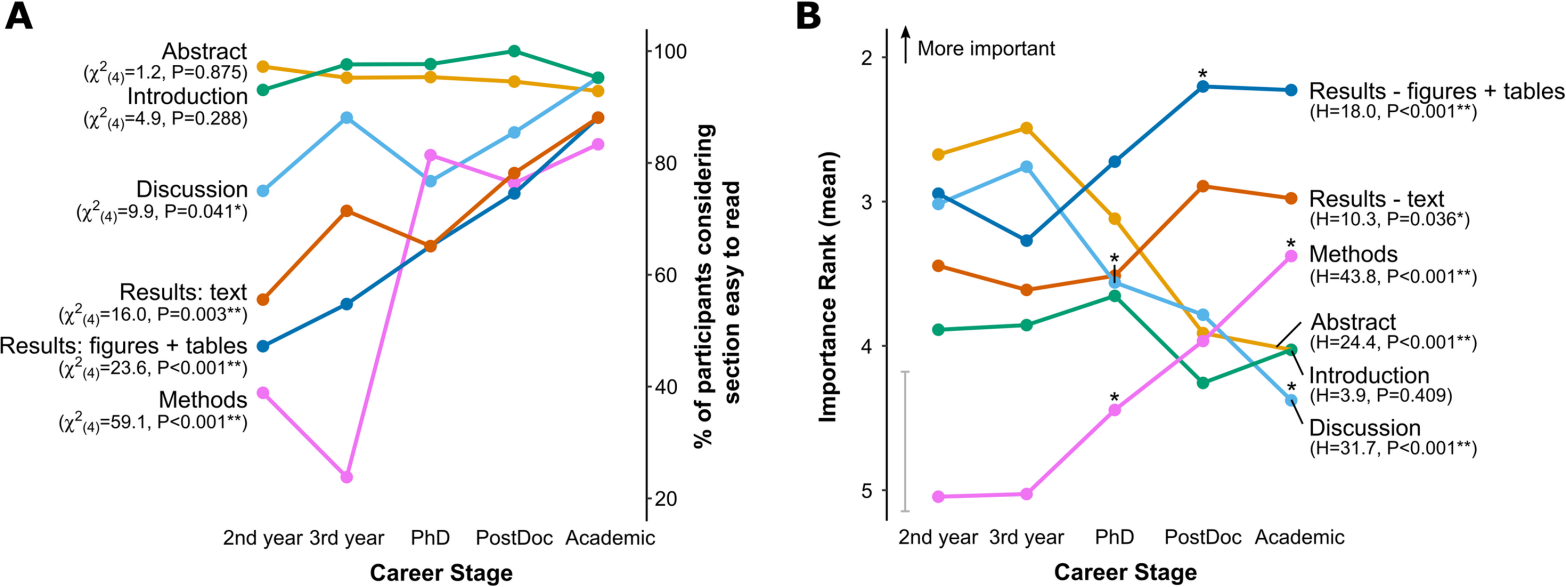
Different sections of scientific papers are considered easy to read and important at different stages of academic careers. A: The proportion of participants considering a section easy to read (presented as ‘Somewhat easy’, ‘easy’ ‘very easy’ combined) as a function of career stage. Results of Chi-square tests are indicated on the left hand side. B: The mean importance rank of sections as a function of career stage. Error bars are omitted from individual points for clarity, with the sole error bar in grey representing the largest 95% confidence interval for any of the data points. Asterisks above data points indicate significant differences in response compared with the previous career stage as determined by Mann-Whitney post-hoc tests.

Nearly all participants considered the Abstract and Introduction sections to be relatively easy to read, with around 90% of all participants of all career stages describing these sections as easy to read (sum of responses to ‘Somewhat easy’, ‘Easy’ or ‘Very Easy’) (Fig 2A). However, there were highly significant differences in the frequencies of academics at different career stages describing the Methods, Results and Discussion sections as easy to read (Methods χ^2^(4) = 59.6, P < 0.001**; Figures χ^2^(4) = 23.6, P <0.001**; Results χ^2^(4) = 16.0, P = 0.003**; Discussion χ^2^(4) = 9.9, P = 0.041*). Fewer than half of undergraduates described the Materials and Methods section as being easy to read (2nd years 29%; 3rd years 24%), in contrast to PhD students and senior researchers (PhD students 81%; PostDocs 76%; Academics 83%). There was a very large shift in responses between 3rd year undergraduates and PhD students, but no significant difference in response was seen for any other career transition. For the results section (both the text and the figures/tables) there were increases in the proportion considering the section easy to read across all career stages. This was particularly striking for Figures and Tables, where 61% of academics described these as being ‘Very Easy’ to understand, compared with only 9% of PostDoctoral researchers.

### Senior researchers place higher value on results and methods sections than those starting their academic careers

To explore the value that researchers placed on different sections of papers, we asked participants to rank the 6 sections in order of how important they thought that section was for understanding the paper, thereby forcing participants to make a relative value judgement (Fig 2B). There were significant differences in the rank given as a function of career stage for all sections with the exception of the introduction section, which was consistently regarded as relatively unimportant. The Abstract was seen as the most important section by undergraduates, whereas the Figures and Tables were ranked as most important to postgraduate researchers. The relative importance rank of the abstract decreased significantly as the level of seniority increased (H = 24.36, d.f. = 4, P <0.001**), with a similar pattern for the discussion (H = 31.75, d.f. = 4, P <0.001*). In contrast, the methods section was rated as the least important by undergraduates, but this section significantly increased in relative importance as a function of career stage (H = 43.77, d.f. = 4, P <0.001**), with academics considering this the 3rd most important section on average. The relative importance of both the text and figures/tables of the results also increased across the career span to become the two most highly ranked sections, with the Figures and Tables being ranked as more important than the text of the Results by all groups. There was therefore a major shift in the relative importance placed on different aspects of papers whereby undergraduate students ranked the abstract and discussion as being the most important sections, but senior researchers saw these as relatively unimportant for understanding the paper. In contrast, experienced researchers saw the Figures and Tables and Results as being the most important sections, but these were relatively undervalued by undergraduates (Fig 2B).

### Researchers at different career stages read papers for different purposes

**Fig 3:**
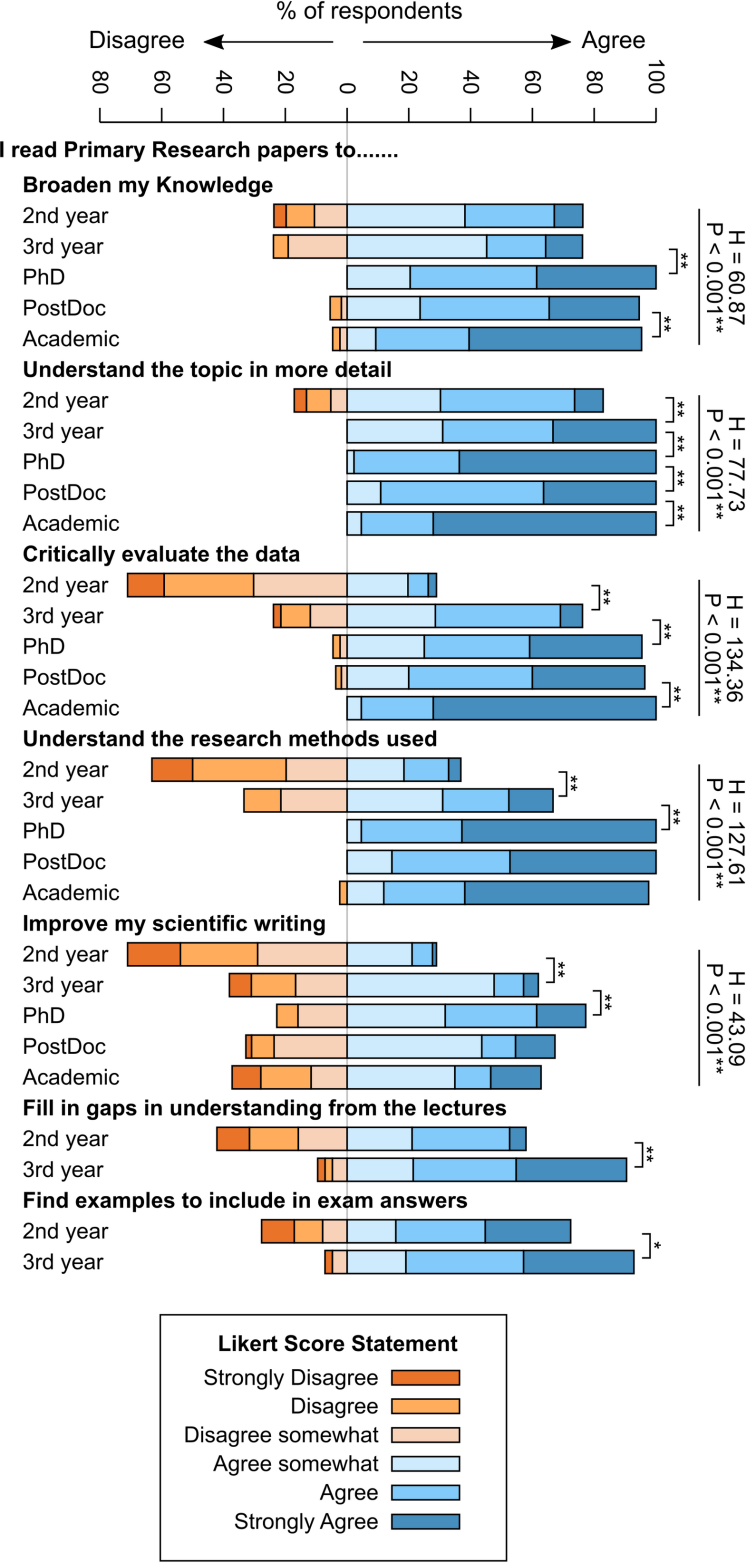
Researchers at different career stages read papers for different purposes. Responses to the 6 point Likert scale questions for 2nd year undergraduate n = 79; 3rd year undergraduate n = 42; PhD student n = 44; PostDoctoral researcher n = 55; Academic n = 43. Statistics alongside straight lines indicate the results of Kruskal-Wallis tests (H and P values presented, degrees of freedom = 4 in all cases). Square brackets indicate significant Mann-Whitney tests for differences between groups, ** indicates P<0.01, * indicates P<0.05.

Scientific researchers read papers for many different reasons, which may shape the relative importance placed on different sections of a manuscript. To explore this, we asked participants to what extent they agreed with a series of 5 statements about the purpose of reading scientific papers, with undergraduates being presented with a further 2 statements relating to the use of papers in relation to their studies. For all questions there were statistically significant differences in the levels of agreement with the statement between respondents at different career stages (Fig 3). The most significant responses were to the statements ‘[I read primary research papers to] critically evaluate the data’ and ‘understand the research methods used’, where undergraduates were significantly less likely to agree with the statement than researchers (Critically evaluate H = 134.36, d.f. = 4, P <0.001*; Research methods H = 127.61, d.f. = 4, P < 0.001*). Interestingly there was also a significant difference in the extent to which PostDocs and Academics agreed with the statement about critical evaluation (U = 715.0, P < 0.001**). 72% of 2nd year undergraduates agreed to some extent with the statement ‘[I read primary research papers to] find examples to include in exam answers’, but only 28% of this group agreed with the statement about critical evaluation. For final year undergraduates this increased to 92% agreement about examples for exams, and 76% for critical evaluation. There were also differences between the sources of papers being read frequently by the participants at different career stages; 16% of 2nd year undergraduates reported reading papers they had found themselves more than once a week, compared with 41% of PhD students and 63% of academics.

### Experienced researchers recommend a selective approach to scientific reading

**Fig 4:**
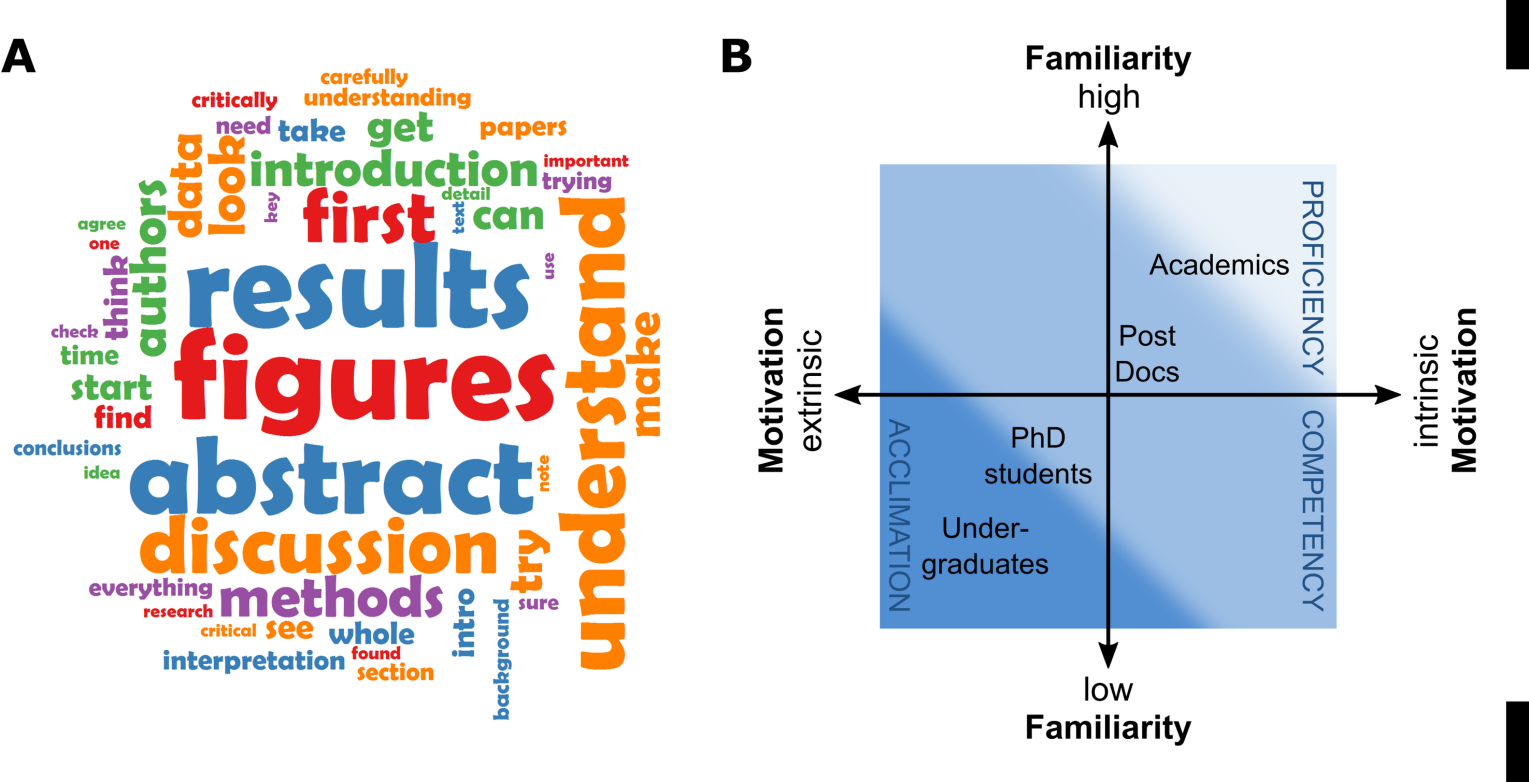
Increased familiarity with scientific literature allows experienced researchers to read papers selectively. A: Wordcloud extracted from advice given by researchers to someone reading a scientific paper for the first time. The size of the word is proportional to the frequency with which the word was mentioned. Words mentioned fewer than 5 times, and the words ‘read’ ‘reading’ and ‘papers’ are excluded for clarity. Colours used do not represent any aspect of analysis. B: Model of development of scientific literature processing skills. Researchers move acclimation through competency to proficiency through their careers, although these should be viewed as a continuum rather than defined phases. This development of skill is associated with increased familiarity with scientific papers, terminology and background knowledge, and with a change from extrinsic to intrinsic motivations for reading.

At the end of the survey, we asked the researchers “If you could only give one piece of advice to someone reading a scientific paper for the first time, what would it be?”, and obtained 88 responses from the 142 participants. To gain an initial impression of the responses, the most commonly used words in the text of the advice given were determined (Fig 4A). Removing the words ‘paper’,‘read’ and ‘reading’, the most commonly used words in the responses were results (n = 28), figures (n = 26) and abstract (n = 25), reflecting the emphasis that senior researchers placed on their own reading of papers (Fig 4A).

A more detailed qualitative analysis of the responses identified four major themes of the advice; reading selectively within a paper, practical suggestions for reading the paper, reading critically and reading with a specific purpose or question in mind (Table 1). 60 of the 88 participants recommended selective reading, with 28 researchers advising reading the paper in a specific order. There was no clear consensus within the responses of which order the sections of a paper should be prioritised in, although starting with the abstract (n = 16) and figures (n = 6) were the most common suggestions. Some researchers recommended reading the abstract, then looking at the figures then reading the text, while others recommended starting with the abstract, then reading the introduction and discussion before looking at the figures. Some recommended prioritising particular sections of the paper (e.g. figures, n = 7) without giving specific instructions on which order the paper should be read in. Others did not make reference to particular sections, but gave more general advice such as identifying the key questions or hypotheses of the paper (n = 8). Some survey participants recommended ignoring the methods section of the paper altogether (n = 3).

**Table 1:** Thematic analysis of advice researchers gave to someone reading a scientific paper for the first time.

Theme (% of participants mentioning advice, n = 88)		
Major theme	Sub-theme(s)	
Read selectively within the paper (68%)	Read sections in a specific order (32%)	
		*Read the abstract first (18%)*
		*Look at the figures first (7%)*
	Prioritise specific sections of the paper (20%)	
		*Prioritise the figures (8%)*
	Identify key ideas in the paper (9%)	

Practical strategies for reading papers (53%)	Read the paper multiple times (20%)	
	Take your time (7%)	
	Use specific sections of the paper to determine if it is worth reading at all (6%)	
	Don’t get bogged down in technical details and/or terminology (6%)	

Read critically (34%)	Assess whether the authors conclusions match the data (15%)	
	Interpret the data for yourself (6%)	
Read the paper with a specific purpose or questions in mind (7%)		

88 respondents gave responses as free text, which were subsequently coded by theme. Main themes (bold) and sub-themes (italics) are presented below, with those being mentioned fewer than 5 times not presented for clarity. Specific sections of papers are mentioned where appropriate. Some responses included multiple pieces of advice.

Other advice related to practical strategies for reading papers, the most common of which was to read the paper multiple times (n = 18), with several researchers advising to read in more detail with each read of the manuscript. 5 researchers highlighted the importance of using the abstract and/or discussion to determine whether a paper was worth reading at all before reading in detail. A related theme was to read with a specific purpose in mind, with 6 researchers advising to be constantly thinking about why the paper is being read, and to read with specific questions in mind. The remaining major theme was to read critically, which was mentioned by 30 of the 88 respondents. Common pieces of advice were to assess whether the conclusions matched the data (n = 18), and to interpret the data for yourself (n = 5).

## Discussion

In this study, we identify significant differences in both opinions of papers and reading strategies between researchers at all career stages within the institution surveyed, indicating that the way researchers engage with scientific literature varies throughout an academic career. This study was originally conceived to investigate differences between undergraduate students and researchers, however subsequent analysis revealed significant differences in responses between PhD students, PostDoctoral researchers and senior academics, which are unlikely to be attributed to further formal training. All participants agreed that reading papers was important for their scientific training. Undergraduates at this research-intensive institution typically read scientific papers to broaden their knowledge and to find examples for exam answers, prioritised reading the abstract and discussion sections, and were most likely to report low confidence in their ability to process the literature. Experienced researchers read papers for a variety of reasons, valued the results and methods sections and were confident in their reading abilities. PhD students and PostDoctoral researchers had intermediate levels of confidence, and PhD students in particular prioritised their reading differently to both undergraduates and experienced researchers. Postgraduates reported a similar ease of reading for both the text and the figures/tables of results sections, whereas undergraduates considered figures and tables more difficult to engage with than the accompanying text, perhaps reflecting the specialist skills needed to interpret graphs and other visual representations of data [20].

Although this study is based on single institution which may not be representative, our data suggest both a change in the motivation for reading through an academic career and an increase in confidence when processing scientific text. 3^rd^ year undergraduates primarily reported reading papers to find examples to find examples for examination answers, which we define as ‘extrinisic’ motivation. This contrasts with experienced researchers who were more likely to report intrinsic motivations for reading. This is reminiscent of goal orientation theories of motivation, where individuals may be either seeking ‘mastery’ of a topic for self-improvement, or demonstration of enhanced ‘performance’ of abilities relative to others [21,22]. Seeking mastery is associated with deep learning approaches and increased self-regulation, which reinforce each other in a positive feedback loop [22]. Conversely, students who are performance motivated may lack the incentive to engage deeply with a topic. Undergraduates motivated primarily by exam performance may therefore adopt a relatively superficial approach to the literature; for students with multiple time pressures this may be highly efficient, but provide poor training for later study or subsequent research careers. Assessment strategies should therefore be considered carefully if the aim is to encourage deep and critical engagement with the literature. Instructors should also be mindful of the low self-efficacy many undergraduate and graduate students have when encountering complex literature, and adopt strategies that increase the confidence of inexperienced readers. It should be noted that undergraduates at this institution are assessed predominantly through terminal written examinations that reward the ability to include examples from scientific literature, but have limited scope for detailed critical analysis of research papers. As such, the nature of the institution and assessment strategy may have influenced engagement with the literature, and participants in our survey may not be representative of those on other courses with alternative assessment models; the relationship between assessment and engagement with literature warrants further research.

Conceptual models describing the development of scientific processing skills have been described previously. One study of literature use amongst Masters level students proposed a scheme based on Models of Domain Learning [23–25] whereby there is a continual acquisition of scientific processing skills, with individuals moving from an initial ‘acclimation’ phase, through to ‘competency’ and to eventual ‘proficiency’ in use of the literature [23–25]. Our data support and extend these models, with the development of literature processing skills being a function of (i) the familiarity with both scientific language and the wider research context and (ii) the degree of intrinsic motivation for reading (Fig 4B). Our data also suggest that the transition from acclimation to proficiency takes place over an extended period of time spanning multiple career stages. Undergraduate students have previously been shown to define conclusions of scientific papers differently to expert readers, and to overlook important grounds for particular conclusions, i.e. were more likely to rely on author statements in the discussion than to interpret primary results for themselves [14]. Inexperienced readers find the language of scientific papers challenging [23], and relative low levels of prior knowledge acts as a barrier to comprehension [26,27]. The importance of background knowledge was specifically commented on by one participant, who added the following disclaimer to their advice for someone reading a paper for the first time (the last question in the survey):

[Participant 83—Academic]

“*This is quite a different question than [earlier ones in the survey]*, *which depend entirely on how familiar I am with the field covered by the paper*. *I answered [the earlier questions] as if the paper were from a field with which I had a lot of familiarity; almost the reverse would be true for a paper outside of my immediate experience*”

This suggests that even experienced researchers adopt more superficial reading strategies when encountering literature outside their area of expertise. This suggests that considerable experience of reading papers cannot substitute for a lack of prior knowledge, and the barrier to deep engagement with literature is the lack of an appropriate framework rather than reading experience *per se*. Our survey did not ask researchers to consider papers of different levels of familiarity, however expert readers adjusting their reading strategy on the basis of prior knowledge is consistent with previous studies [28]. Experienced readers reading within their field therefore benefit from having developed a ‘purpose-laden schema’ which allows the new information to be processed within their existing framework of knowledge [29], as well as a personal research interest to motivate their reading. This contrasts significantly with undergraduate students, who lack a personal research context and prior knowledge to relate their reading to; in our opinion it is therefore unrealistic to expect students to engage with the literature in the same way as professional researchers. Instructors should therefore be mindful of the lack of subject competency that inexperienced readers have to contextualise their reading, and ensure readers are introduced to relevant background concepts in more accessible formats before tackling highly technical literature.

Given the challenges of scientific language, lack of prior knowledge and low self-confidence, there is a need to support the development of academic reading and scientific processing skills within degree programmes. A variety of teaching strategies for introducing papers undergraduate students have been described, many of which have been demonstrated to increase student confidence and skill in the use of research literature [6,11,15,16,30–32]. A commonly used model is the CREATE method, which suggests structuring reading as Consider, then Read, Elucidate the hypothesis, Analyse and interpret data and then to Think of the next Experiment [11]. In doing so this approach models the research process, therefore may provide undergraduates with sufficient personal research context to adopt a reading strategy closer to that typically used by experienced researchers. Our data also suggest that PhD students and PostDoctoral researchers may benefit from structured reading support, as individuals are unlikely have mastered this skill by the end of an undergraduate programme. Reading to support personal research may also require graduate students to adapt strategies used when they were undergraduates, therefore specific guidance in using the literature would be appropriate to support this transition. Focussed training on appropriate use of research papers should therefore be incorporated in postgraduate programmes, and activities promoting critical engagement with the literature should be embedded in the day-to-day activities of research groups. Research supervisors should also be mindful that saying “go and read a paper” may be interpreted differently by individuals at different career stages, so should be clear in their instructions and expectations.

A broader issue for the research community is highlighted in our data, in that even experienced researchers can find the scientific literature challenging. 42% of academics surveyed found reading papers frustrating, despite seeing it as a valuable use of their time. The frustrations researchers have with reading primary papers are sometimes discussed in an ironic or humorous manner [33–35], reflecting the widespread difficulties that many have with this form of scientific communication. Although we did not explore the specific causes of these frustrations in greater detail, previous studies and our interactions with both students and colleagues suggest that background knowledge, terminology, generic attributes of scientific papers and unfamiliar techniques are the most challenging aspects of papers for inexperienced readers [23]. These difficulties may be particularly acute for those reading scientific literature in a second language, and for those with Specific Learning Differences such as dyslexia [36]. Those from disadvantaged backgrounds may also have lower levels of academic language proficiency and confidence with complex text [37]. Individuals with lower self confidence or self-efficacy are less likely to adopt deep learning approaches and evaluate information critically [38], so may also need structured support to engage with the literature fully.

It should be noted that this study is based on participants at a research intensive university, most of whom who have chosen to embark on a scientific career. They therefore represent a self-selecting sample who may have been more confident with reading technical literature than peers who left research, therefore raising the possibility that research literature is itself a barrier to scientific career choices. In addition, our model of a constantly developing engagement with scientific literature is based on participants at a single institution. We only received responses from ~45% of the undergraduate population and were unable to determine the response rate from researchers, so our results may not be representative of all individuals at the institution and should therefore be treated with caution. It will be important to investigate whether these initial findings hold true for researchers from different career stages at other institutions in the UK and beyond; we hope that this study will provoke others to consider this largely unexplored difficulty with written scientific communication. In a world of open access publishing scientific papers are reaching wider audiences [39]; readers may include researchers from other fields unfamiliar with discipline specific vocabulary, as well as the general public who may struggle with the complexities of scientific text. As science becomes an increasingly data rich environment, and interdisciplinary approaches to research questions expand, the skills to efficiently process new scientific literature are only going to become more valuable. We therefore propose that the scientific community considers the language they are using to communicate their findings, and how this could be made more accessible and inclusive in order to benefit non-scientists and scientists across all career stages.

## Supporting information

S1 File Copy of the student survey questions. This survey was administered online via www.surveymonkey.com, so the layout presented does not match the online version. The text and order of questions is identical, with the exception of where institutional specific terms have been amended for a broader audience [indicated with italic text].
(PDF)

S2 File Copy of the researcher survey questions. This survey was administered online via www.surveymonkey.com, so the layout presented does not match the online version, however the text and order of questions is identical.
(PDF)

S3 File Raw data from the survey responses collected online. (XLSX)

S4 File Disciplinary backgrounds of survey participants. For A: 2nd year undergraduates and B: 3rd year undergraduates, participants were asked which courses they were taking a proxy for disciplinary background. For C: Researcher, participants were asked ‘Which of the following best describes your area of research?’ and were able to select multiple options. Response rates cannot be determined for researchers as surveys were distributed via departmental bulletins or email lists for which the total number of individuals contacted is unknown.
(PDF)

## References

[1] Phillips LM, Norris SP. Bridging the gap between the language of science and the language of school science through the use of adapted primary literature. Research in Science Education. 2009;39: 313–319.

[2] Tenopir C, King DW. Engineering Communication Patterns Compared with Science and Medicine. Communication Patterns of Engineers. John Wiley & Sons, Inc.; 2003 pp. 149–161.

[3] Tenopir C, King DW, Edwards S, Wu L. Electronic journals and changes in scholarly article seeking and reading patterns. Aslib Proc. 2009;61: 5–32.

[4] Snow CE, Johnson J, Martin-Hansen L, Schleppegrell MJ, Fang Z, Coxhead A, et al Academic Language and the Challenge of Reading for Learning About Science. Science. 2010;328: 12–452. 10.1126/science.1182597 20413488

[5] Fang Z. Scientific literacy: A systemic functional linguistics perspective. Sci Educ. Wiley Subscription Services, Inc., A Wiley Company; 2005;89: 335–347.

[6] Round JE, Campbell AM. Figure facts: Encouraging undergraduates to take a data-centered approach to reading primary literature. CBE Life Sci Educ. 2013;12: 39–46. doi: 10.1187/cbe.11-07-0057 23463227 10.1187/cbe.11-07-0057 PMC3587854

[7] The Quality Assurance Agency for Higher Education. Subject Benchmark Statement UK Quality Code for Higher Education [Internet]. Gloucester: The Quality Assurance Agency for Higher Education; 2015 Available: http://www.qaa.ac.uk/en/Publications/Documents/SBS-Biosciences-15.pdf

[8] Dunbar S. Dealing with arsenic–an investigation with undergraduates–Plant Science Today [Internet]. 2 Dec 2015 [cited 21 Jul 2017]. Available: http://blog.aspb.org/2015/12/02/dealing-with-arsenic-an-investigation-with-undergraduates/

[9] Thompson K. How to read a journal article [Internet]. Royal Society of Chemistry; Available: http://www.rsc.org/learn-chemistry/resource/res00001653/how-to-read-a-journal-article?cmpid=CMP00004937

[10] Williams M. How to Read a Scientific Paper [Internet]. American Society of Plant Biologists; 2016 Available: http://aspb.org/wp-content/uploads/2016/04/HowtoReadScientificPaper.pdf

[11] Hoskins SG, Stevens LM, Nehm RH. Selective use of the primary literature transforms the classroom into a virtual laboratory. Genetics. 2007;176: 1381–1389. doi: 10.1534/genetics.107.071183 17483426 10.1534/genetics.107.071183 PMC1931557

[12] Willmott CJR, Clark RP, Harrison TM. Introducing undergraduate students to scientific reports. Bioscience Education. The Higher Education Academy Innovation Way, York Science Park, Heslington, York YO10 5BR; 2003;1 doi: 10.3108/beej.2003.01010010

[13] Spiegelberg BD. A focused assignment encouraging deep reading in undergraduate biochemistry. Biochem Mol Biol Educ. 2014;42: 1–5. doi: 10.1002/bmb.20744 24243802 10.1002/bmb.20744

[14] van Lacum E, Ossevoort M, Buikema H, Goedhart M. First Experiences with Reading Primary Literature by Undergraduate Life Science Students. Int J Sci Educ. 2012;34: 1795–1821.

[15] Hoskins SG, Lopatto D, Stevens LM. The C.R.E.A.T.E. approach to primary literature shifts undergraduates’ self-assessed ability to read and analyze journal articles, attitudes about science, and epistemological beliefs. CBE Life Sci Educ. 2011;10: 368–378. doi: 10.1187/cbe.11-03-0027 22135371 10.1187/cbe.11-03-0027 PMC3228655

[16] Kozeracki CA, Carey MF, Colicelli J, Levis-Fitzgerald M, Grossel M. An intensive primary-literature-based teaching program directly benefits undergraduate science majors and facilitates their transition to doctoral programs. CBE Life Sci Educ. 2006;5: 340–347. doi: 10.1187/cbe.06-02-0144 17146041 10.1187/cbe.06-02-0144 PMC1681356

[17] Sato BK, Kadandale P, He W, Murata PMN, Latif Y, Warschauer M. Practice makes pretty good: Assessment of primary literature reading abilities across multiple large-enrollment biology laboratory courses. CBE Life Sci Educ. 2014;13: 677–686. doi: 10.1187/cbe.14-02-0025 25452490 10.1187/cbe.14-02-0025 PMC4255354

[18] Coil D, Wenderoth MP, Cunningham M, Dirks C. Teaching the process of science: faculty perceptions and an effective methodology. CBE Life Sci Educ. 2010;9: 524–535. doi: 10.1187/cbe.10-01-0005 21123699 10.1187/cbe.10-01-0005 PMC2995770

[19] Bransford JD, Brown AL, Cocking RR. How People Learn: Brain, Mind, Experience and School. National Academy Press; 2000.

[20] Glazer N. Challenges with graph interpretation: a review of the literature. Studies in Science Education. 2011;47: 183–210.

[21] Dweck CS, Leggett E. A social-cognitive approach to motivation and personality. Psychol Rev. 1988;95: 256–273.

[22] De Clercq M, Galand B, Frenay M. Chicken or the egg: Longitudinal analysis of the causal dilemma between goal orientation, self-regulation and cognitive processing strategies in higher education. Studies in Educational Evaluation. 2013;39: 4–13.

[23] Lie R, Abdullah C, He W, Tour E. Perceived Challenges in Primary Literature in a Master’s Class: Effects of Experience and Instruction. CBE Life Sci Educ. American Society for Cell Biology; 2016;15 doi: 10.1187/cbe.15-09-0198 27909027 10.1187/cbe.15-09-0198 PMC5132374

[24] Alexander PA. The Development of Expertise: The Journey From Acclimation to Proficiency. Educ Res. American Educational Research Association; 2003;32: 10–14.

[25] Alexander PA. Mapping the multidimensional nature of domain learning: The interplay of cognitive, motivational, and strategic forces In: Maehr M L Pintrich P, editor. Advances in motivation and achievement. Greenwich, CT: JAI Press; 1997 pp. 213–250.

[26] Brill G, Falk H, Yarden A. The learning processes of two high-school biology students when reading primary literature. Int J Sci Educ. 2004;19: 497–512.

[27] Ozuru Y, Dempsey K, McNamara DS. Prior knowledge, reading skill, and text cohesion in the comprehension of science texts. Learning and Instruction. 2009;19: 228–242.

[28] Bazerman C. Shaping Written Knowledge: The Genre and Activity of the Experimental Article in Science. Madison: University of Wisconsin Press; 1988.

[29] Bazerman C. Physicists Reading Physics. Written Communication. SAGE Publications Inc; 1985;2: 3–23.

[30] Stevens LM, Hoskins SG. The CREATE strategy for intensive analysis of primary literature can be used effectively by newly trained faculty to produce multiple gains in diverse students. CBE Life Sci Educ. 2014;13: 224–242. doi: 10.1187/cbe.13-12-0239 26086655 10.1187/cbe.13-12-0239 PMC4041501

[31] Koeneman M, Goedhart M, Ossevoort M. Introducing Pre-university Students to Primary Scientific Literature Through Argumentation Analysis. Research in Science Education. 2013;43: 2009–2034.

[32] Cook LK, Mayer RE. Teaching readers about the structure of scientific text. Journal of Educational Psychology. 1988;80: 448.

[33] Cham J. PhD comics: Anxiety vs. Reading. In: PhD comics [Internet]. 1 Mar 2002 [cited 21 Jul 2017]. Available: http://phdcomics.com/comics/archive.php?comicid=287

[34] Cham J. PhD comics: Journal Club, pt. 2. In: PhD comics [Internet]. 1 Nov 2008 [cited 21 Jul 2017]. Available: http://phdcomics.com/comics/archive.php?comicid=963

[35] Ruben A. How to read a scientific paper. In: Science | AAAS [Internet]. 20 Jan 2016 [cited 19 Jul 2017]. Available: http://www.sciencemag.org/careers/2016/01/how-read-scientific-paper

[36] Mortimore T, Crozier WR. Dyslexia and difficulties with study skills in higher education. Studies in Higher Education. Routledge; 2006;31: 235–251.

[37] Murray N. Widening participation and English language proficiency: a convergence with implications for assessment practices in higher education. Studies in Higher Education. Routledge; 2013;38: 299–311.

[38] Abouserie R. Self-esteem and achievement motivation as determinants of students’ approaches to studying. Studies in Higher Education. 1995;20: 19–26.

[39] Davis PM, Lewenstein BV, Simon DH, Booth JG, Connolly MJL. Open access publishing, article downloads, and citations: randomised controlled trial. BMJ. 2008;337: a568 doi: 10.1136/bmj.a568 18669565 10.1136/bmj.a568 PMC2492576

