# Supplementary material for: Perceptions of scientific research literature and strategies for reading papers depend on academic career stage
Source: PLoS One. 2017 Dec 28;12(12):e0189753. doi: 10.1371/journal.pone.0189753 (PMC5746228; doi:10.1371/journal.pone.0189753)
Supplement: S1 File — This survey was administered online via www.surveymonkey.com, so the layout presented does not match the online version. The text and order of questions is identical, with the exception of where institutional specific terms have been amended for a broader audience [indicated with italic text]. (PDF) [file pone.0189753.s001.pdf]

S1 File: Undergraduate Student Survey

NB This survey was administered online via www.surveymonkey.com, so the layout below does not match the online version. The text and order of questions is identical, with the exception of where institutional specific terms have been amended for a broader audience [indicated with italic text].

**1: Which year of study are you in?**	
2nd year	*3rd year*

**2: Which subjects did you study for your *2nd year*?**			
Animal Biology	Biochemistry	Cell and Developmental Biology	Ecology
Neurobiology	Pathology	Pharmacology	Physiology
Plant and Microbial Sciences	Other [please specify]		

**3: Which *3rd year* subject are you studying?**			
I am a *2nd year* student	Biochemistry	Genetics	Neuroscience
Pathology	Pharmacology	Physiology, Neuroscience and Development	Plant Sciences
Zoology	Other [please specify]		

**4: On average, how many PRIMARY RESEARCH papers do you read?**					
Papers recommended by lecturers	None	One per term	One per lecture block	One per week	More than one per week
Papers required for *tutorial* discussions	None	One per term	One per lecture block	One per week	More than one per week
Papers recommended by *tutors*	None	One per term	One per lecture block	One per week	More than one per week
Papers recommended by other students	None	One per term	One per lecture block	One per week	More than one per week
Papers you have found yourself	None	One per term	One per lecture block	One per week	More than one per week

**5: On average, how many REVIEW papers do you read?**					
Papers recommended by lecturers	None	One per term	One per lecture block	One per week	More than one per week
Papers required for *tutorial* discussions	None	One per term	One per lecture block	One per week	More than one per week
Papers recommended by *tutors*	None	One per term	One per lecture block	One per week	More than one per week
Papers recommended by other students	None	One per term	One per lecture block	One per week	More than one per week
Papers you have found yourself	None	One per term	One per lecture block	One per week	More than one per week

**6: When trying to finding a paper, which of the following tools do you use? Select all that apply.**			
Google	Google Scholar	PubMed	Mendeley
ScienceDirect	Web of Science	Scopus	Direct links to papers provided by others
Direct links to papers obtained via social media (e.g. Twitter, Facebook)	Other (please specify)		

**7: To what extent would you agree with the following statements?**						
I enjoy reading research papers	Strongly Disagree	Disagree	Disagree somewhat	Agree somewhat	Agree	Strongly Agree
I am confident in reading research papers without guidance (e.g. from supervisors)	Strongly Disagree	Disagree	Disagree somewhat	Agree somewhat	Agree	Strongly Agree
I know how to find research papers on a given topic	Strongly Disagree	Disagree	Disagree somewhat	Agree somewhat	Agree	Strongly Agree
I know how to read research papers to extract information efficiently	Strongly Disagree	Disagree	Disagree somewhat	Agree somewhat	Agree	Strongly Agree
I know how to identify papers that are of critical importance to the area I am investigating	Strongly Disagree	Disagree	Disagree somewhat	Agree somewhat	Agree	Strongly Agree

**8: To what extent would you agree with the following statements?**						
Reading research papers is a good use of my time	Strongly Disagree	Disagree	Disagree somewhat	Agree somewhat	Agree	Strongly Agree
Reading research papers is frustrating	Strongly Disagree	Disagree	Disagree somewhat	Agree somewhat	Agree	Strongly Agree
Reading research papers is important for my general scientific training	Strongly Disagree	Disagree	Disagree somewhat	Agree somewhat	Agree	Strongly Agree
Reading research papers is important for success in this years exams	Strongly Disagree	Disagree	Disagree somewhat	Agree somewhat	Agree	Strongly Agree

**9: To what extent would you agree with the following statements:<br>I read PRIMARY RESEARCH papers to .....**						
Broaden my knowledge	Strongly Disagree	Disagree	Disagree somewhat	Agree somewhat	Agree	Strongly Agree
Understand the topic in more detail	Strongly Disagree	Disagree	Disagree somewhat	Agree somewhat	Agree	Strongly Agree
Critically evaluate the data	Strongly Disagree	Disagree	Disagree somewhat	Agree somewhat	Agree	Strongly Agree
Fill in gaps in understanding from the lectures	Strongly Disagree	Disagree	Disagree somewhat	Agree somewhat	Agree	Strongly Agree
Find examples to include in exam answers	Strongly Disagree	Disagree	Disagree somewhat	Agree somewhat	Agree	Strongly Agree
Understand the research methods used	Strongly Disagree	Disagree	Disagree somewhat	Agree somewhat	Agree	Strongly Agree

Improve my scientific writing	Strongly Disagree	Disagree	Disagree somewhat	Agree somewhat	Agree	Strongly Agree
Other (please specify)						

**10: To what extent would you agree with the following statements: I read REVIEW papers to .....**						
Broaden my knowledge	Strongly Disagree	Disagree	Disagree somewhat	Agree somewhat	Agree	Strongly Agree
Understand the topic in more detail	Strongly Disagree	Disagree	Disagree somewhat	Agree somewhat	Agree	Strongly Agree
Critically evaluate the data	Strongly Disagree	Disagree	Disagree somewhat	Agree somewhat	Agree	Strongly Agree
Fill in gaps in understanding from the lectures	Strongly Disagree	Disagree	Disagree somewhat	Agree somewhat	Agree	Strongly Agree
Find examples to include in exam answers	Strongly Disagree	Disagree	Disagree somewhat	Agree somewhat	Agree	Strongly Agree
Understand the research methods used	Strongly Disagree	Disagree	Disagree somewhat	Agree somewhat	Agree	Strongly Agree
Improve my scientific writing	Strongly Disagree	Disagree	Disagree somewhat	Agree somewhat	Agree	Strongly Agree
Other (please specify)						

**11: How easy do you usually find it to understand the following aspects of a research paper?**						
Abstract	Very Difficult	Difficult	Somewhat difficult	Somewhat easy	Easy	Very Easy
Introduction	Very Difficult	Difficult	Somewhat difficult	Somewhat easy	Easy	Very Easy
Materials and Methods	Very Difficult	Difficult	Somewhat difficult	Somewhat easy	Easy	Very Easy
Results – Figures and Tables	Very Difficult	Difficult	Somewhat difficult	Somewhat easy	Easy	Very Easy
Results – Text based description	Very Difficult	Difficult	Somewhat difficult	Somewhat easy	Easy	Very Easy
Discussion	Very Difficult	Difficult	Somewhat difficult	Somewhat easy	Easy	Very Easy

**12: How important do you think the following aspects of a research paper are for understanding it?**						
Abstract	Very Unimportant	Unimportant	Somewhat unimportant	Somewhat important	Important	Very Important
Introduction	Very Unimportant	Unimportant	Somewhat unimportant	Somewhat important	Important	Very Important
Materials and Methods	Very Unimportant	Unimportant	Somewhat unimportant	Somewhat important	Important	Very Important
Results – Figures and Tables	Very Unimportant	Unimportant	Somewhat unimportant	Somewhat important	Important	Very Important
Results – Text based description	Very Unimportant	Unimportant	Somewhat unimportant	Somewhat important	Important	Very Important
Discussion	Very Unimportant	Unimportant	Somewhat unimportant	Somewhat important	Important	Very Important

**13: Please rank the following sections of research papers in the order of how easy you find them to read. 1 = easiest, 6 = most difficult.**					
Abstract	Introduction	Materials and Methods	Results – Figures and Tables	Results – Text based description	Discussion

**14: Please rank the following sections of papers in the order of importance you think they are to understanding research papers. 1 = most important, 6 = least important.**					
Abstract	Introduction	Materials and Methods	Results – Figures and Tables	Results – Text based description	Discussion

[Teaching Questions not relevant to current manuscript have been omitted for clarity here]

**15: I give my consent for my answers to this survey to be used in any publications resulting from this work**	
Yes	No
