# Supplementary material for: Perceptions of scientific research literature and strategies for reading papers depend on academic career stage
Source: PLoS One. 2017 Dec 28;12(12):e0189753. doi: 10.1371/journal.pone.0189753 (PMC5746228; doi:10.1371/journal.pone.0189753)
Supplement: S2 File — This survey was administered online via www.surveymonkey.com, so the layout presented does not match the online version, however the text and order of questions is identical. (PDF) [file pone.0189753.s002.pdf]

S2 File: Researcher Survey

NB This survey was administered online via www.surveymonkey.com, so the layout below does not match the online version, however the text is identical.

**1: Which of the following best describes you?**			
PhD student	PostDoctoral Researcher	Head of Group/UTO	Other [please specify]

**2: Which department do you work in?**			
Biochemistry	Psychology	Genetics	Pathology
Pharmacology	Physiology, Neuroscience and Development (PDN)	Plant Sciences	Zoology

**3: Which of the following best describes your area of research? Select all that apply**			
Ecology	Evolutionary Biology	Physiology	Cell Biology
Molecular Biology	Genetics	Biochemistry	Systems Biology
Mathematical Biology	Other [please specify]		

**4: Have you done any undergraduate teaching in the last three years?**	
Yes	No

[Teaching Questions not relevant to current manuscript have been omitted for clarity here]

**5: On average, how many PRIMARY RESEARCH papers do you read?**						
Papers recommended by research supervisor	Not applicable	None	One every 3 months	One per week	One per day	More than one per day
Papers recommended by colleagues	Not applicable	None	One every 3 months	One per week	One per day	More than one per day
Papers you have found yourself	Not applicable	None	One every 3 months	One per week	One per day	More than one per day

**6: On average, how many REVIEW papers do you read?**						
Papers recommended by research supervisor	Not applicable	None	One every 3 months	One per week	One per day	More than one per day
Papers recommended by colleagues	Not applicable	None	One every 3 months	One per week	One per day	More than one per day
Papers you have found yourself	Not applicable	None	One every 3 months	One per week	One per day	More than one per day

**7: When trying to finding a paper, which of the following tools do you use? Select all that apply.**			
Google	Google Scholar	PubMed	Mendeley
ScienceDirect	Web of Science	Scopus	Direct links to papers provided by others
Direct links to papers obtained via social media (e.g. Twitter, Facebook)	Other (please specify)		

**8: To what extent would you agree with the following statements?**						
I enjoy reading research papers	Strongly Disagree	Disagree	Disagree somewhat	Agree somewhat	Agree	Strongly Agree
I am confident in reading research papers without guidance (e.g. from supervisors)	Strongly Disagree	Disagree	Disagree somewhat	Agree somewhat	Agree	Strongly Agree
I know how to find research papers on a given topic	Strongly Disagree	Disagree	Disagree somewhat	Agree somewhat	Agree	Strongly Agree
I know how to read research papers to extract information efficiently	Strongly Disagree	Disagree	Disagree somewhat	Agree somewhat	Agree	Strongly Agree
I know how to identify papers that are of critical importance to the area I am investigating	Strongly Disagree	Disagree	Disagree somewhat	Agree somewhat	Agree	Strongly Agree

**9: To what extent would you agree with the following statements?**						
Reading research papers is a good use of my time	Strongly Disagree	Disagree	Disagree somewhat	Agree somewhat	Agree	Strongly Agree
Reading research papers is frustrating	Strongly Disagree	Disagree	Disagree somewhat	Agree somewhat	Agree	Strongly Agree
Reading research papers is important for my general scientific training	Strongly Disagree	Disagree	Disagree somewhat	Agree somewhat	Agree	Strongly Agree

**10: To what extent would you agree with the following statements:**<br>**I read PRIMARY RESEARCH papers to .....**						
Broaden my knowledge	Strongly Disagree	Disagree	Disagree somewhat	Agree somewhat	Agree	Strongly Agree
Understand the topic in more detail	Strongly Disagree	Disagree	Disagree somewhat	Agree somewhat	Agree	Strongly Agree
Critically evaluate the data	Strongly Disagree	Disagree	Disagree somewhat	Agree somewhat	Agree	Strongly Agree
Understand the research methods used	Strongly Disagree	Disagree	Disagree somewhat	Agree somewhat	Agree	Strongly Agree
Improve my scientific writing	Strongly Disagree	Disagree	Disagree somewhat	Agree somewhat	Agree	Strongly Agree
Other (please specify)						

**11: To what extent would you agree with the following statements: I read REVIEW papers to .....**						
Broaden my knowledge	Strongly Disagree	Disagree	Disagree somewhat	Agree somewhat	Agree	Strongly Agree
Understand the topic in more detail	Strongly Disagree	Disagree	Disagree somewhat	Agree somewhat	Agree	Strongly Agree
Critically evaluate the data	Strongly Disagree	Disagree	Disagree somewhat	Agree somewhat	Agree	Strongly Agree
Understand the research methods used	Strongly Disagree	Disagree	Disagree somewhat	Agree somewhat	Agree	Strongly Agree
Improve my scientific writing	Strongly Disagree	Disagree	Disagree somewhat	Agree somewhat	Agree	Strongly Agree
Other (please specify)						

**12: How easy do you usually find it to understand the following aspects of a research paper?**						
Abstract	Very Difficult	Difficult	Somewhat difficult	Somewhat easy	Easy	Very Easy
Introduction	Very Difficult	Difficult	Somewhat difficult	Somewhat easy	Easy	Very Easy
Materials and Methods	Very Difficult	Difficult	Somewhat difficult	Somewhat easy	Easy	Very Easy
Results – Figures and Tables	Very Difficult	Difficult	Somewhat difficult	Somewhat easy	Easy	Very Easy
Results – Text based description	Very Difficult	Difficult	Somewhat difficult	Somewhat easy	Easy	Very Easy
Discussion	Very Difficult	Difficult	Somewhat difficult	Somewhat easy	Easy	Very Easy

**13: How important do you think the following aspects of a research paper are for understanding it?**						
Abstract	Very Unimportant	Unimportant	Somewhat unimportant	Somewhat important	Important	Very Important
Introduction	Very Unimportant	Unimportant	Somewhat unimportant	Somewhat important	Important	Very Important
Materials and Methods	Very Unimportant	Unimportant	Somewhat unimportant	Somewhat important	Important	Very Important
Results – Figures and Tables	Very Unimportant	Unimportant	Somewhat unimportant	Somewhat important	Important	Very Important
Results – Text based description	Very Unimportant	Unimportant	Somewhat unimportant	Somewhat important	Important	Very Important
Discussion	Very Unimportant	Unimportant	Somewhat unimportant	Somewhat important	Important	Very Important

**14: Please rank the following sections of research papers in the order of how easy you find them to read. 1 = easiest, 6 = most difficult.**					
Abstract	Introduction	Materials and Methods	Results – Figures and Tables	Results – Text based description	Discussion

**15: Please rank the following sections of papers in the order of importance you think they are to understanding research papers. 1 = most important, 6 = least important.**					
Abstract	Introduction	Materials and Methods	Results – Figures and Tables	Results – Text based description	Discussion

**16: If you could only give one piece of advice to someone reading a scientific paper for the first time, what would it be?**
**17: If you have any comments on how you use the scientific literature please write these in the box below:**

**18: I give my consent for my answers to this survey to be used in any publications resulting from this work**	
Yes	No
