# Supplementary material for: Perceptions of scientific research literature and strategies for reading papers depend on academic career stage
Source: PLoS One. 2017 Dec 28;12(12):e0189753. doi: 10.1371/journal.pone.0189753 (PMC5746228; doi:10.1371/journal.pone.0189753)
Supplement: S4 File — For A: 2nd year undergraduates and B: 3rd year undergraduates, participants were asked which courses they were taking a proxy for disciplinary background. For C: Researcher, participants were asked ‘Which of the following best describes your area of research?’ and were able to select multiple options. Response rates cannot be determined for researchers as surveys were distributed via departmental bulletins or email lists for which the total number of individuals contacted is unknown. (PDF) [file pone.0189753.s004.pdf]

A

2nd year Undergraduates		
Students take 3 courses from the options below:	No of participants	% of participants
Pathology	37	46
Cell and Developmental Biology	34	42
Plant and Microbial Sciences	29	36
Biochemistry and Molecular Biology	27	33
Neurobiology	27	33
Other	22	27
Pharmacology	21	26
Physiology	19	23
Animal Biology	14	17
Ecology	7	9
Experimental Psychology	6	7
**Total number of survey participants**	**81**	
**Total number of 2nd year undergraduates**	**174**	
**Response rate**	**47%**	

B

3rd year Undergraduates		
Students take 1 course from the options below:	No of participants	% of participants
Plant Sciences	17	26
Biochemistry	15	23
Zoology	15	23
Pathology	5	8
Pharmacology	5	8
Physiology, Neuroscience and Development	5	8
Genetics	3	5
Neuroscience	1	2
**Total number of survey participants**	**66**	
**Total number of 3rd year undergraduates**	**156**	
**Response rate**	**42%**	

C

Researchers	PhD student		Post Doctoral Researcher		Academic		Total	
Note that some participants selected multiple options from the following:	No of participants	% of participants	No of participants	% of participants	No of participants	% of participants	No of participants	% of participants
Molecular Biology	17	32	25	37	13	27	55	32
Cell Biology	9	17	12	18	13	27	34	20
Genetics	14	26	12	18	6	12	32	19
Ecology	13	25	11	16	5	10	29	17
Other	9	17	5	7	14	29	28	16
Evolutionary Biology	6	11	12	18	8	16	26	15
Biochemistry	10	19	9	13	6	12	25	15
Physiology	8	15	6	9	10	20	24	14
Systems Biology	3	6	5	7	5	10	13	8
Mathematical Biology	2	4	6	9	4	8	12	7
**Total number of researchers**	**53**		**68**		**49**		**170**	

S4 File: Disciplinary Backgrounds of survey participants. For **A:** 2nd year undergraduates and **B:** 3rd year undergraduates, participants were asked which courses they were taking a proxy for disciplinary background. Response rates were calculated as a proportion of students on biological sciences courses. For **C:** Researcher, participants were asked 'Which of the following best describes your area of research?' and were able to select multiple options. Response rates cannot be determined for researchers as surveys were distributed via departmental bulletins or email lists for which the total number of individuals contacted is unknown.
